# 

**Published:** 1909-09

**Authors:** 


					CONTENTS.
Page
Referred Cardiac Pain.
By F. G. Thomson, B.A. Cantab., M.D. Lond.
193
Tuberculosis of the Colon.
By Eknest W. Hey Groves, M.S., B.Sc.,
F.R.C.S.
203
Theoretical Considerations of Pulmonary Percussion Notes.
By
George Hely-Hutchinson Almond, M.A., M.ts. (Jxon.
215
Some Points in the Technique of the Radical Operation for
Chronic Otitis Media Purulenta.
By P. Watson Williams,
M D. Lond.
224
Sleep and the Modern Hypnotics.
By J. M. Fortescue-Brickdale,
M.A., M.D. Oxon.
230
The Safetv of the Patient in Anaesthesia.
By John Freeman,
M.D. Durh., F.R.C.S. Edin.
238
Progress of the Medical Sciences :
Medicine.
J. R. Charles, M.A., M.D., B.C. Cantab.
240
Surgery.
Harold F. Mole, F.R.C.S. ...
243
Reviews of Books
247
Editorial Notes
269
Notes on Preparations for the Sick
279
The Library of the Bristol Medico-Chirurgical Society   282
Local Medical Notes
284

				

